# Recent development and biomedical applications of probabilistic Boolean networks

**DOI:** 10.1186/1478-811X-11-46

**Published:** 2013-07-01

**Authors:** Panuwat Trairatphisan, Andrzej Mizera, Jun Pang, Alexandru Adrian Tantar, Jochen Schneider, Thomas Sauter

**Affiliations:** 1 Life Sciences Research Unit, University of Luxembourg, Luxembourg; 2 Computer Science and Communications Research Unit, University of Luxembourg, Luxembourg; 3 Luxembourg Centre for Systems Biomedicine, University of Luxembourg, Luxembourg; 4 Interdisciplinary Centre for Security, Reliability and Trust, University of Luxembourg, Luxembourg; 5 Saarland University Medical Center, Department of Internal Medicine II, Homburg, Saarland, Germany

**Keywords:** Probabilistic Boolean networks, Probabilistic graphical models, Qualitative modelling, Systems biology

## Abstract

Probabilistic Boolean network (PBN) modelling is a semi-quantitative approach widely used for the study of the topology and dynamic aspects of biological systems. The combined use of rule-based representation and probability makes PBN appealing for large-scale modelling of biological networks where degrees of uncertainty need to be considered.

A considerable expansion of our knowledge in the field of theoretical research on PBN can be observed over the past few years, with a focus on network inference, network intervention and control. With respect to areas of applications, PBN is mainly used for the study of gene regulatory networks though with an increasing emergence in signal transduction, metabolic, and also physiological networks. At the same time, a number of computational tools, facilitating the modelling and analysis of PBNs, are continuously developed.

A concise yet comprehensive review of the state-of-the-art on PBN modelling is offered in this article, including a comparative discussion on PBN versus similar models with respect to concepts and biomedical applications. Due to their many advantages, we consider PBN to stand as a suitable modelling framework for the description and analysis of complex biological systems, ranging from molecular to physiological levels.

## Background

A large number of formal representation types that exist in Systems Biology are used to construct distinctive mathematical models, each with their own strengths and weaknesses. On one hand, deciphering the complexity of biological systems by quantitative methods, such as ordinary differential equation (ODE) based mathematical models, yields detailed representations with high predictive power. Such an approach is however often hampered by the low availability and/or identifiability of kinetic parameters and experimental data [1]. These limitations often result in the generation of relatively small quantitative network models. On the other hand, qualitative modelling frameworks such as the Boolean Networks (BNs), allow for describing large biological networks while still preserving important properties of the systems [2]. The models pertaining to this latter class fail nevertheless to offer a quantitative determination of the system’s dynamics due to their inherent qualitative nature.

Probabilistic Boolean networks (PBNs) were introduced in 2002 by Shmulevich et al. as an extension of the Boolean Network concept and as an alternative for modelling gene regulatory networks [3]. PBNs combine the rule-based modelling of a BN, as introduced by Kauffman [4-7], with uncertainty principles, e.g., as described by a Markov chain [8]. In terms of applications, analogously to the case of traditional BNs, the qualitative nature of state and time in a PBN framework allows for modelling of large-scale networks. The integrated stochastic properties of PBNs additionally enable semi-quantitative properties to be extracted. Existing analytic methods on PBNs allow for gaining a better understanding of how biological systems behave, and offer in addition the means to compare to traditional BNs. Examples are the calculation of influences which represent the quantitative strength of interaction between certain genes [3], or the determination of steady-state distributions to quantitatively predict the activity of certain genes in steady state [8].

It has been shown in the past years that the use of PBNs in the biological field is not limited to the molecular level, but also can potentially be linked to applications in clinic. To name a few, Tay et al. constructed a PBN to demonstrate the interplay between dengue virus and different cytokines which mediate the course of disease in dengue haemorrhagic fever (DHF) [9]. Ma et al. processed functional Magnetic Resonance Imaging (fMRI) signals to infer a brain connectivity network comparing between Parkinson’s disease patients and healthy subjects [10]. Even though the research efforts on PBNs in this direction are just sprouting, the results from such PBN studies can provide a first clue on a disease’s etiology and progression. As PBNs are highly flexible for data integration and as there exist a number of computational tools for PBN analysis, PBN is a suitable modelling approach to integrate information and derive knowledge from omic scale data which should in turn facilitate a physician’s decision-making process in clinic.

For the past decade, PBNs were the object of extensive studies, both theoretical and applied. Among theoretical topics, there are steady-state distribution, e.g., [11-13], network construction and inference, e.g., [14-16], network intervention and control, e.g., [17-19]. Several minor topics were investigated as well, including reachability analysis [20] or sensitivity analysis [21]. Other studies dealt with PBNs in biological systems at multi-level such as gene regulatory networks [22-24], signal transduction networks [25], metabolic networks [26], and also physiological networks [9,10] which could potentially link to medicine as previously mentioned. In parallel, a number of computational tools which facilitate the modelling and analysis of PBNs are also continuously developed [27-29]. Given the continuous development in this area due to the broad on-going range of research on PBNs, we offer a state-of-the-art overview on this modelling framework. A comparison of PBN to other graphical probabilistic modelling approaches is also enclosed, specifically with respect to Bayesian networks. Last but not least, a view of the theoretical and applied research on PBNs as models for the study of multi-level biomedical networks is included.

In order to provide a coherent overview of the recent advances on PBN, we start with several theoretical aspects, organised as follows: an introduction to PBNs and associated dynamics are given in Section ‘Introduction to probabilistic Boolean networks and their dynamics’, the construction and inference of PBNs as models for gene regulatory networks are presented in Section ‘Construction and inference of PBNs as models of gene regulatory networks’, structural intervention and external control are discussed in Section ‘Structural intervention and control of PBNs’, ending with the relationship between PBNs and other probabilistic graphical models in Section ‘Relationship between PBNs and other probabilistic graphical models’. Later, in Section ‘PBN applications in biological and biomedical studies’ we present a broad summary of PBN applications as a representation of biological networks followed by a discussion on the future applications of PBN in Systems Biology and Systems Biomedicine. A short conclusion is given in Section ‘Conclusion’.

## Introduction to probabilistic Boolean networks and their dynamics

### Boolean networks

A *Boolean Network* (BN) *G*(*V*,*F*), as originally introduced by Kauffman [4-7], is defined as a set of binary-valued variables (nodes) *V* = {*x*_1_,*x*_2_,…,*x*_*n*_} and a vector of Boolean functions ***f*** = (*f*_1_,…,*f*_*n*_). At each updating epoch, referred to as time point *t* (*t* = 0,1,2,…), the *state* of the network is defined by the vector ***x***(*t*) = (*x*_1_(*t*),*x*_2_(*t*),…,*x*_*n*_(*t*)), where *x*_*i*_(*t*) is the value of variable *x*_*i*_ at time *t*, i.e., *x*_*i*_(*t*) ∈ {0,1} (*i* = 1,2,…,*n*). For each variable *x*_*i*_ there exists a *predictor set*$\left\{x_{i_{1}},x_{i_{2}},\ldots ,x_{i_{k(i)}}\right\}$ and a Boolean *predictor function* (or simply *predictor*) *f*_*i*_ being the *i*-th element of ***f*** that determines the value of *x*_*i*_ at the next time point, i.e.,

(1) $$x_{i}(t+1)=f_{i}(x_{i_{1}}(t),x_{i_{2}}(t),\ldots ,x_{i_{k(i)}}(t)),$$

where 1 ≤ *i*_1_ < *i*_2_ < ⋯ < *i*_*k*(*i*)_ ≤ *n*. Since the predictor functions of ***f*** are time-homogenous, the notation can be simplified by writing $f_{i}(x_{i_{1}},x_{i_{2}},\ldots ,x_{i_{k(i)}})$. Without loss of generality, *k*(*i*) can be defined to be a constant equal to *n* for all *i* by introducing *fictitious* variables in each function: the variable *x*_*i*_ is fictitious for a function *f* if *f*(*x*_1_,…,*x*_*i*−1_,0,*x*_*i*+1_,…,*x*_*n*_) = *f*(*x*_1_,…,*x*_*i*−1_,1,*x*_*i*+1_,…,*x*_*n*_) for all possible values of *x*_1_,…,*x*_*i*−1_,*x*_*i*+1_,…,*x*_*n*_. A variable that is not fictitious is referred to as *essential*. The *k*(*i*) elements of the predictor set $\left\{x_{i_{1}},x_{i_{2}},\ldots ,x_{i_{k(i)}}\right\}$ are referred to as the *essential predictors* of variable *x*_*i*_. The vector ***f*** of predictor functions constitutes the *network transition function* (or simply the *network function*). The network function ***f*** determines the time evolution of the states of the Boolean network, i.e., ***x***(*t*+1) = ***f***(***x***(*t*)). Thus, the BN’s dynamics is deterministic. The only potential uncertainty is in the selection of the initial starting state of the network.

**Figure 1:**
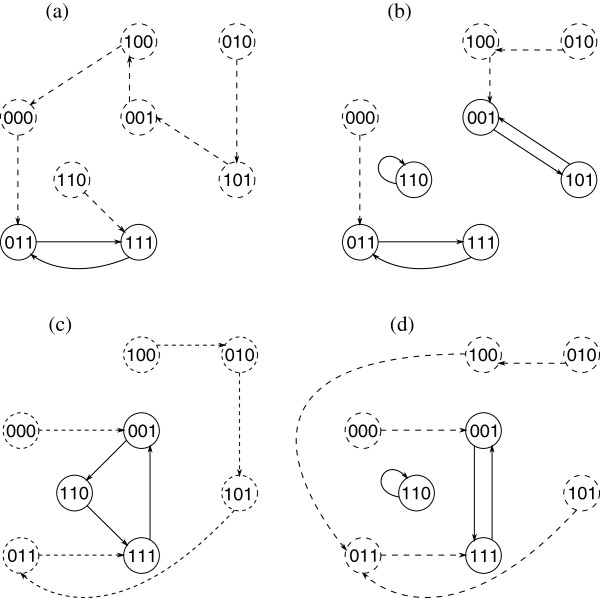
**State transition diagrams of the four constituent Boolean networks of the PBN in Figure**2**.** For each constituent BN the attractor states and the transitions between them are indicated with solid circles and arrows, respectively. The remaining transitions and transient states are indicated with dashed arrows and circles, respectively. **(a)** The constituent BN of the PBN in Figure 2 corresponding to transition function ***f***_1_. There is only one attractor, i.e., {011,111}, which is a cyclic attractor. **(b)** The constituent BN of the PBN in Figure 2 corresponding to transition function ***f***_2_. There are two cyclic attractors: {011,111}, {001,101} and one singleton attractor: {110}. **(c)** The constituent BN of the PBN in Figure 2 corresponding to transition function ***f***_3_. {001,110,111} is the cyclic attractor. **(d)** The constituent BN of the PBN in Figure 2 corresponding to transition function ***f***_4_. There are two attractors: a cyclic one, i.e., {001,111} and a singleton one, i.e., {110}.

Given an initial state, within a finite number of steps, the BN will transition into a fixed state or a set of states through which it will repeatedly cycle forever. In the first case, each such fixed state is called a *singleton attractor*, whereas in the second case, the set of states is referred to as a *cyclic attractor*. An *attractor* is either a singleton or a cyclic attractor. The number of transitions required to return to a given state in an attractor is the *cycle length* of that attractor. The *attractor structure* of the BN is determined by the particular combination of singleton and cyclic attractors, and by the cycle lengths of the cyclic attractors. The states within an attractor are called *attractor states*. Non-attractor states are called *transient* and are visited at most once on any network trajectory. The states that lead into an attractor constitute its *basin of attraction*. The basins form a partition of the state space of the BN. For example, in Figure 1 the state transition diagrams of four different Boolean networks with three variables are given (in fact all these Boolean networks constitute a probabilistic Boolean network — the framework of probabilistic Boolean networks is presented in Section ‘5’). For each of these networks attractor states and transient states are indicated and the cyclic- and singleton attractors are given.

A *Boolean Network with perturbations* (BNp) is a BN with an introduced positive probability for which, at any transition, the network can depart from its current trajectory into a randomly chosen state, which becomes an initial state of a new trajectory. Formally, the perturbation mechanism is modelled by introducing a parameter *p*, 0 < *p* < 1, and a so-called *perturbation vector****γ*** = (*γ*_1_,*γ*_2_,…,*γ*_*n*_), where *γ*_1_,*γ*_2_,…,*γ*_*n*_ are independent and identically distributed (i.i.d.) binary-valued random variables ^a^ such that Pr{*γ*_*i*_ = 1} = *p*, and Pr{*γ*_*i*_ = 0} = 1−*p*, for all *i*=1,2,…,*n*. For every transition step of the network a new realisation of the perturbation vector is given. If ***x***(*t*) ∈ {0,1}^*n*^ is the state of the network at time *t*, then the next state ***x***(*t* + 1) is given by either ***f***(***x***(*t*)) or by ***x***(*t*) ⊕ ***γ***(*t*), where ⊕ is component-wise addition modulo 2 and ***γ***(*t*) ∈ {0,1}^*n*^ is the realisation of the perturbation vector for the current transition. The choice of the state transition rule depends on the current realisation of the perturbation vector. Two cases are distinguished: either ***γ***(*t*) = **0** or at least one component of ***γ***(*t*) is 1, i.e., ***γ***(*t*) ≠ **0**. In the first case, which happens with probability (1−*p*)^*n*^, the next state is given by ***f***(***x***(*t*)). In the second case, given with probability 1−(1−*p*)^*n*^, the next state is determined as ***x***(*t*) ⊕ ***γ***(*t*): if *γ*_*i*_ = 1, then *x*_*i*_ changes its value; otherwise it does not (*i* = 1,2,…,*n*). Since ***γ***(*t*) ≠ **0**, at least one of the nodes flips its value.

The attractors of a Boolean network characterise its long-run behaviour [8]. However, if random perturbations are incorporated, the network can escape the attractors. In particular, perturbations allow the system to reach any of its states from any current state in one transition. In consequence, the dynamics of the BNp is given by an *ergodic* Markov chain [30], ^b^ having a unique stationary distribution which simultaneously is its steady-state (limiting) distribution. The steady-state probability distribution, where each state is assigned a non-zero probability, characterises the long-run behaviour of the BNp. Nevertheless, if perturbation probability is very small, the network will remain in the attractors of the original network for most of the time, meaning that attractor states will carry most of the steady-state probability mass [8]. In this way the attractor states remain significant for the description of the long-run behaviour of a Boolean network after adding perturbations. Thus, a BNp inherits the attractor-basin structure from the original BN; however, once an attractor has been reached, the network remains in it until a perturbation occurs that throws the network out of it [31].

### Probabilistic Boolean networks

PBNs were introduced in order to overcome the deterministic rigidity of BNs [3,32,33], originally as a model for gene regulatory networks. A PBN consists of a finite collection of BNs, each defined by a fixed network function, and a probability distribution that governs the switching between these BNs.

Formally, a probabilistic Boolean network G(V,F) is defined by a set of binary-valued variables (nodes)^c^*V* = {*x*_1_,*x*_2_,…,*x*_*n*_} and a list of sets $F=(F_{1},F_{2},\ldots ,F_{n})$. For *i* = 1,2,…,*n* the set *f*_*i*_ is given as $\left\{f_{1}^{\left(i\right)},f_{2}^{\left(i\right)},\ldots ,f_{l(i)}^{\left(i\right)}\right\}$, where $f_{j}^{\left(i\right)}$, 1 ≤ *j* ≤ *l*(*i*), is a possible Boolean predictor function for the variable *x*_*i*_, with *l*(*i*) the number of possible predictors for *x*_*i*_. In general, each node *x*_*i*_ can have *l*(*i*) different sets of essential predictors, each specified for a particular predictor function in *f*_*i*_. A *realisation* of the PBN at a given instant of time is determined by a vector of predictor functions, where the *i*th element of that vector contains the function selected at that time point for *x*_*i*_. For a PBN with *N* realisations there are *N* possible network transition functions ***f***_1_,***f***_2_,…,***f***_*N*_ of the form $f_{l}=(f_{l_{1}}^{\left(1\right)},f_{l_{2}}^{\left(2\right)},\ldots ,f_{l_{n}}^{\left(n\right)})$, *l* = 1,2,…,*N*, 1 ≤ *l*_*j*_ ≤ *l*(*j*), $f_{l_{j}}^{\left(j\right)}\in F_{j}$, and *j* = 1,2,…,*n*. Each network function ***f***_*l*_ defines a constituent Boolean network, or *context*, of the PBN.

Let ***f*** = (*f*^(1)^,*f*^(2)^,…,*f*^(*n*)^) be a random vector taking values in *F*_1_ × *F*_2_ × ⋯ × *F*_*n*_; in other words, ***f*** is a random vector that acquires as value any of the realisations of the PBN. The probability that the predictor $f_{j}^{\left(i\right)}$, 1 ≤ *j* ≤ *l*(*i*), is selected to determine the value of *x*_*i*_ is given by

(2) $$c_{j}^{\left(i\right)}=\text{Pr}\{f^{\left(i\right)}=f_{j}^{\left(i\right)}\}=\underset{l:f_{l_{i}}^{\left(i\right)}=f_{j}^{\left(i\right)}}{\sum }\text{Pr}\{f=f_{l}\}.$$

**Figure 2:**
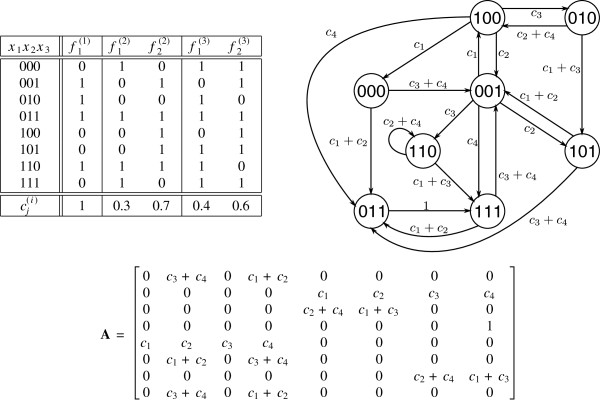
**An example of truth table, state transition diagram, and transition probability matrix of a PBN.** The truth table, the state transition diagram, and the transition probability matrix **A** of a PBN without perturbations consisting of three variables *V* = {*x*_1_,*x*_2_,*x*_3_} and $F=(F_{1},F_{2},F_{3})$, where $F_{1}=\{f_{1}^{\left(1\right)}\}$, $F_{2}=\{f_{1}^{\left(2\right)},f_{2}^{\left(2\right)}\}$, and $F_{3}=\{f_{1}^{\left(3\right)},f_{2}^{\left(3\right)}\}$. Since there is one predictor function for node *x*_1_ and two predictors for nodes *x*_2_ and *x*_3_, there are 1 · 2 · 2 = 4 realisations of the PBN given by four network transition functions $f_{1}=(f_{1}^{\left(1\right)},f_{1}^{\left(2\right)},f_{1}^{\left(3\right)})$, $f_{2}=(f_{1}^{\left(1\right)},f_{1}^{\left(2\right)},f_{2}^{\left(3\right)})$, $f_{3}=(f_{1}^{\left(1\right)},f_{2}^{\left(2\right)},f_{1}^{\left(3\right)})$, and $f_{4}=(f_{1}^{\left(1\right)},f_{2}^{\left(2\right)},f_{2}^{\left(3\right)})$ with associated probabilities *c*_1_ = 0.12, *c*_2_ = 0.18, *c*_3_ = 0.28, and *c*_4_ = 0.42, respectively. For example, $c_{3}=c_{1}^{\left(1\right)}\cdot c_{2}^{\left(2\right)}\cdot c_{1}^{\left(3\right)}=1\cdot 0.7\cdot 0.4=0.28$. The edges in the state transition diagram are labelled with the transition probabilities. As can be seen from the state transition diagram, the underlying Markov chain is irreducible and aperiodic, thus ergodic. The steady-state (limiting) distribution for the chosen *c*_*i*_ values, *i* = 1..4, is given by $\left[\frac{7}{1609},\frac{3640}{14481},\frac{49}{4827},\frac{716}{4827},\frac{175}{4827},\frac{238}{4827},\frac{2548}{14481},\frac{4696}{14481}\right]$ (the states are considered in the lexicographical order from 000 to 111).

It follows that $\overset{l(i)}{\underset{j=1}{\sum }}c_{j}^{\left(i\right)}=1$. The PBN is said to be *independent* if the random variables *f*^(1)^,*f*^(2)^,…,*f*^(*n*)^ are independent. Assuming independence, there are $N=\overset{n}{\underset{i=1}{\prod }}l(i)$ realisations (constituent BNs) of the PBN and the probability distribution on ***f*** governing the selection of a particular realisation is given by $\text{Pr}\{f=f_{l}\}=\overset{n}{\underset{i=1}{\prod }}c_{l_{i}}^{\left(i\right)}$. An example of a PBN with three nodes is given in Figure 2.

At each time point of the PBN’s evolution, a decision is made whether to switch the constituent network. This is modelled with a binary random variable *ξ* : if *ξ* = 0, then the current constituent network is preserved; if *ξ* = 1, then a context is randomly selected from all the constituent networks in accordance with the probability distribution of ***f***. Notice that this definition implies that there are two mutually exclusive ways in which the context may remain unchanged: 1) either *ξ* = 0 or 2) *ξ* = 1 and the current network is reselected. The *functional switching* probability *q* = Pr(*ξ* = 1) is a system parameter. Two cases are distinguished in the literature: if *q* = 1, then a switch is made at each updating epoch; if *q* < 1, then the PBN’s evolution in consecutive time points proceeds in accordance with a given constituent BN until the random variable *ξ* calls for a switch. If *q* = 1, as originally introduced in [32], the PBN is said to be *instantaneously random*; if *q* < 1, it is said to be *context-sensitive*. The former models uncertainty in model selection, the latter models the situation where the model is affected by latent variables outside the model [34]. As an example let us consider the PBN given in Figure 2. Let the PBN be instantaneously random, i.e., *q* = 1. The four constituent BNs associated with the four transition functions ***f***_1_, ***f***_2_, ***f***_3_, and ***f***_4_, are given in Figure 1. Further, let us assume that the initial state is the state 101 and that the consecutive realisations are ***f***_1_,***f***_2_,***f***_4_,***f***_3_,***f***_2_,***f***_2_,***f***_3_,***f***_4_,***f***_4_,…. Then, the corresponding time evolution of the PBN (trajectory) is given by the following sequence of state transitions: 101 → 001 → 110 → 110 → 111 → 011 → 111 → 001 → 100 → 011 → …. Irrespective of which constituent network (realisation) is selected next, the consecutive state in the trajectory is going to be 111 as the probability of moving from 011 to 111 is *c*_1_ + *c*_2_ + *c*_3_ + *c*_4_ = 1.

A *Probabilistic Boolean Network with perturbations* (PBNp) is the variant of the PBN framework in which each constituent network is a BNp with a common perturbation probability parameter *p*, 0 < *p* < 1, and a perturbation vector ***γ***. If ***x***(*t*) ∈ {0,1}^*n*^ is the current state of the network and ***γ***(*t*) = **0**, then the next state of the network is determined according to the current network function ***f***_*l*_, i.e., ***x***(*t*+1) = ***f***_*l*_(***x***(*t*)). If ***x***(*t*) ∈ {0,1}^*n*^ is the current state and ***γ***(*t*) ≠ **0**, then ***x***(*t*+1) = ***x***(*t*) ⊕ ***γ***(*t*). Whereas a context switch in a PBNp corresponds to a change in latent variables, resulting in a structural change in the functions that govern the PBNp, a random perturbation reflects a transient value change that leaves the network wiring unmodified, as for example in the case of gene activation or inactivation caused by external stimuli such as stress conditions or small molecule inhibitors [8].

**Figure 3:**
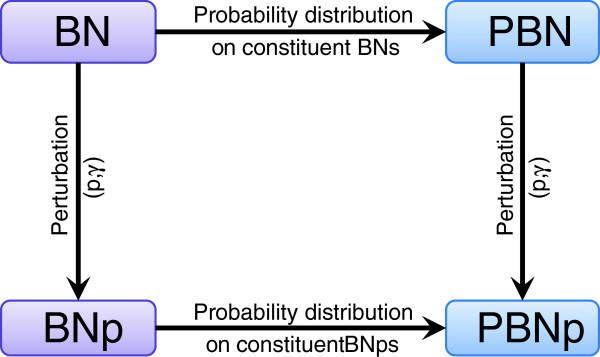
**Relationships between the frameworks of Boolean and probabilistic Boolean networks.** A Boolean network (BN) can be converted to a Boolean network with perturbations (BNp) by introducing a probability parameter *p*, 0 < *p* < 1, and a perturbation vector (***γ***). A probabilistic Boolean network (PBN) is built upon a number of constituent BNs and a probability distribution governing the choice of the Boolean network in accordance with which the next transition is made. Analogically, a PBN can be converted to a probabilistic Boolean network with perturbations (PBNp) by introducing a probability parameter *p*, 0 < *p* < 1, and a perturbation vector (***γ***). A probabilistic Boolean network (PBN) is built upon a number of constituent BNps and a probability distribution governing the choice of the BNp in accordance with which the next transition is made.

The relationship between the four frameworks, i.e., Boolean networks, Boolean networks with perturbations, probabilistic Boolean networks, and probabilistic Boolean networks with perturbations is schematically depicted in Figure 3.

### Dynamics of PBNs

A Boolean network with perturbations can be viewed as a homogenous irreducible Markov chain ***X***_*t*_, with state space $X={\left\{0,1\right\}}^{n}$, where *n* is the number of nodes in the BNp. Let $P_{y}(x)=\text{Pr}[X_{t_{0}+1}=x|X_{t_{0}}=y]$ be the Markov chain transition probability from state ***y*** to state ***x*** at any instant *t*_0_. This probability is a weighted sum of two transition probabilities, one for the BN, with probability (1−*p*)^*n*^, and the other for the perturbations, with probability 1−(1−*p*)^*n*^, i.e.,

(3) $$P_{y}(x)=1_{\left[f(y)=x\right]}{\left(1-p\right)}^{n}+(1-1_{\left[x=y\right]})p^{\eta (x,y)}{\left(1-p\right)}^{n-\eta (x,y)},$$

where *p* is the perturbation probability, **1** is the indicator function (**1**_[*P*]_ = 1 if the proposition *P* is true, and **1**_[*P*]_ = 0 otherwise), and *η*(***x***,***y***) is the Hamming distance between the binary vectors ***x*** and ***y***.

The Markov chain ***X***_*t*_ is *ergodic*, which follows from the fact that it is aperiodic, irreducible, and defined on a finite state space. In other words, it possesses a unique stationary distribution, being simultaneously its steady-state (limiting) distribution. If $P_{y}^{\left(t\right)}(x)$ is the probability that the system transitions from ***y*** to ***x*** in *t* time steps, i.e., $P_{y}^{\left(t\right)}(x)=\text{Pr}[X_{t_{0}+t}=x|X_{t_{0}}=y]$, then the steady-state distribution *π* of ***X***_*t*_ is defined by $\pi (x)=\underset{t\to \infty }{\lim}P_{k}^{\left(t\right)}(x)$ for any initial state k∈X. For a set of states B⊆X the steady-state probability is given by $\pi (B)=\underset{x\in B}{\sum }\pi (x)=\underset{t\to \infty }{\lim}P_{k}^{\left(t\right)}(B)$ for any initial state k∈X. For example, the steady-state distribution of the Markov chain given by the transition probability matrix in Figure 2 is $\left[\frac{7}{1609},\frac{3640}{14481},\frac{49}{4827},\frac{716}{4827},\frac{175}{4827},\frac{238}{4827},\frac{2548}{14481},\frac{4696}{14481}\right]$ (the states are considered in the lexicographical order from 000 to 111).

In the case of a probabilistic Boolean network, the transition probabilities P_***y***_(***x***) of the underlying Markov chain ***X***_*t*_ depend on the probability of selecting a network transition function ***f***_*k*_, *k* = 1,2,…,*N*, that determines the transition from ***y*** to ***x*** i.e.,

(4) $$P_{y}(x)=\text{Pr}[X_{t+1}=x|X_{t}=y]=\overset{N}{\underset{k=1}{\sum }}1_{\left[f_{k}(y)=x\right]}\cdot \text{Pr}\{f=f_{k}\},$$

where *N*, as before, is the number of constituent BNs and ***f*** is a random vector determining the PBN’s realisation. Letting ***x*** and ***y*** range all states in X, the transition probability matrix **A** of size 2^*n*^ × 2^*n*^ can be formed and expressed as

(5) $$A=\overset{N}{\underset{k=1}{\sum }}A_{k}\cdot \text{Pr}\{f=f_{k}\},$$

where **A**_*k*_ is the transition matrix corresponding to the *k*-th constituent BN.

Now, adding perturbations with probability *p* makes the underlying finite-space Markov chain ***X***_*t*_ of the PBNp aperiodic and irreducible, hence ergodic. This allows the network dynamics of a PBNp to be studied with the use of the rich theory of ergodic Markov chains [30]. In particular, in the case of instantaneously random PBNps, the transition probability matrix $\overset{\sim }{A}$ is given by

(6) $$\tilde{A}={\left(1-p\right)}^{n}\cdot A+\tilde{P},$$

where $\tilde{P}$ is the perturbation matrix of the form

(7) $${\tilde{P}}_{y,x}=(1-1_{\left[x=y\right]})p^{\eta (x,y)}{\left(1-p\right)}^{n-\eta (x,y)},$$

where, as before, **1** is the indicator function and *η* is the Hamming distance. As in the case of BNps, the ergodicy of the underlying Markov chain ensures the existence of the unique stationary distribution being the limiting distribution of the chain.

By definition, the set of attractors of a PBN is the union of the sets of attractors of the constituent networks [8]. Notice that whereas in a BN two attractors cannot intersect, attractors from different contexts can intersect in the case of a PBN. Similarly as in the case of Boolean networks, attractors play a major role in the characterisation of the long-run behaviour of a probabilistic Boolean network. If, however, perturbations are incorporated, the long-run behaviour of the network is characterised by its steady-state distribution. Nevertheless, if both the switching and perturbation probabilities are very small, then the attractors still carry most of the steady-state probability mass [8]. From a biological point of view attractors of such networks are interesting as they can be given a clear biological interpretation: they can be used to model cellular states [31]. For example, in the context of gene regulatory networks, it is believed that attractors can be interpreted as cellular phenotypes [7,8]. Thus, the long-run behaviour of the network given by its steady-state probabilities is of a special interest. Specifically, the attractor steady-state probabilities, i.e., *π*(*A*), where *A* is an attractor, are important. There are a number of approaches towards the determination and analysis of the steady-state distribution of a PBNp. We review them shortly.

First, one approach to the steady-state analysis is to construct the state transition matrix in some form or another and then apply some numerical methods, e.g., iterative, decompositional or projection methods [35]. A transition matrix based approach in which the sparse transition matrix is constructed in an efficient way and the so-called power method, which is applied to compute the steady-state probability distribution, is proposed in [36]. Unfortunately, the size of the state space grows exponentially in the number of nodes (genes) and becomes prohibitive for matrix-based numerical analysis of larger networks [11]. In [12], an approximation method for computing the steady-state probability distribution of a PBNp is derived from the approach of [36]. This method neglects some constituent BNps with very small probabilities during the construction of the transition probability matrix. An error analysis is given to demonstrate the effectiveness of this approach. Further, in [13] and [37] a matrix perturbation method for computing the steady-state probability distribution of PBNps is proposed together with its approximation variant. The proposed methods make use of certain properties of the perturbation matrix, $\tilde{P}$.

**Figure 4:**
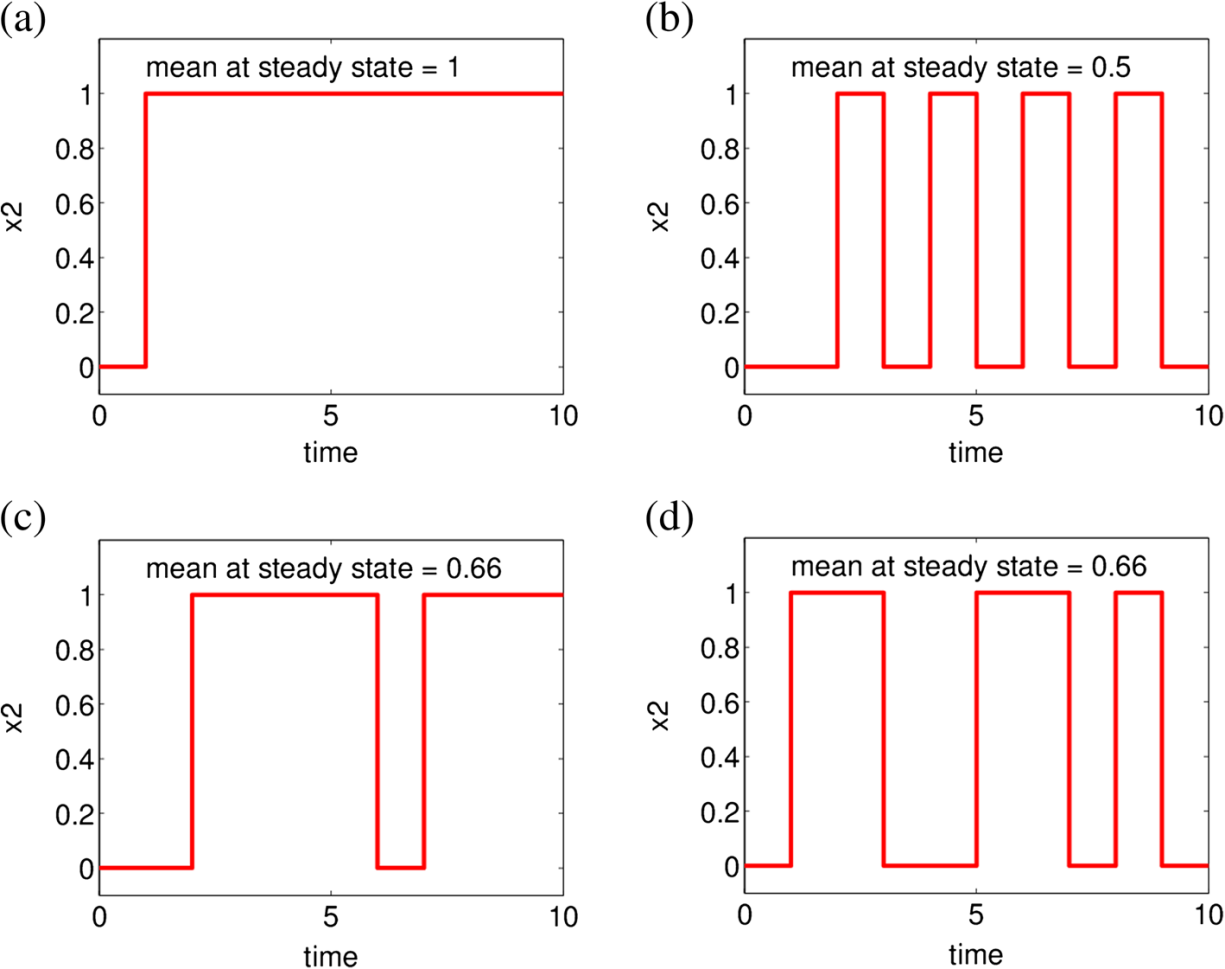
**Dynamical simulations of node** ***x***_**2**_ **of the example network in Figure**2**, with initial state** ***k*** **= 000. (a)** Dynamics of *x*_2_ governed by the constituent BN corresponding to the transition function ***f***_1_, where *c*_1_ = 1, *c*_2_ = *c*_3_ = *c*_4_ = 0. Starting from 000 the periodic attractor {011,111} is reached. The probability of {*x*_2_ = 1} given by the stationary distribution is 1. **(b)** Dynamics of *x*_2_ governed by the constituent BN corresponding to the transition function ***f***_4_, where *c*_4_ = 1, *c*_1_ = *c*_2_ = *c*_3_ = 0. Starting from 000 the periodic attractor {001,111} is reached. The probability of {*x*_2_ = 1} given by the stationary distribution related to the reached attractor, i.e., $\left[0,\frac{1}{2},0,0,0,0,0,\frac{1}{2}\right]$ (the states are considered in the lexicographical order), is 0.5. **(c,d)** Examples of *x*_2_ dynamics in the full PBN as given in Figure 2. Starting from 000 different trajectories are obtained for different simulation runs. The underlying Markov chain is ergodic and a unique stationary distribution, being the steady state (limiting) distribution, exists therefore. The steady state probability of {*x*_2_ = 1} is 0.66.

Second, Markov chain Monte Carlo methods [38] represent a feasible alternative to numerical matrix-based methods for obtaining steady-state distributions. Given an ergodic Markov chain, a Monte Carlo simulation method has been proposed: the probability of being in state ***x*** in the long run can be estimated empirically by simulating the network for a sufficiently long time and by counting the percentage of time the chain spends in that state regardless of the starting state [8]. A set of examples of Monte Carlo simulations from the PBN example in Figure 2 is shown in Figure 4. However, the question that remains is how to judge whether the simulation time is sufficiently long? The key factor here is the convergence, which in the case of a PBNp is known to depend to a large extent on the perturbation probability *p*[11]. Several approaches for determining the number of iterations necessary to achieve convergence were developed. A typical class consists of methods based on the second-largest eigenvalue of the transitions probability matrix, but due to reasons already mentioned above, these approaches can be impractical for larger networks. Another method utilises the so-called *minorisation condition* for Markov chains [39] to provide *a priori* bounds on the number of iterations. However, the usefulness of this approach is also limited (see [11] for details). There exist a number of methods for empirically diagnosing convergence to the steady-state distribution [40,41]. In [11] two of them are considered: one, based on the Kolmogorov-Smirnov test, a nonparametric test for the equality of continuous, one-dimensional probability distributions, and, second, the approach proposed in [42] which reduces the study of convergence of the chain to the investigation of the convergence of a two-state Markov chain. For illustration of application of these approaches to PBNs, we refer to [11] where the joint steady-state probabilities of combinations between two genes in human glioma gene expression data set were analysed.

Finally, as shown in [31], analytical expressions for the attractor steady-state probabilities can be derived both for BNps and PBNps. The obtained formulas are further exploited to propose an approximate steady-state computation algorithm.

We just shortly mention here that in the case of probabilistic Boolean networks without perturbations the dynamics is given by a Markov chain that does not necessarily be ergodic, specifically the Markov chain may contain more than one so-called *ergodic set of states*, also referred to as a closed, irreducible set of states in the literature. An ergodic set of states *C* in a Markov chain is defined as a set of states where all states communicate and no state outside *C* is reachable from any state in *C*^d^. The notion of an ergodic set of the corresponding Markov chain in probabilistic Boolean networks is the stochastic analogue of the notion of an attractor in standard Boolean networks [32]. Notice, however, that the ergodic sets and the attractors of a PBN or PBNp may differ. In the case of probabilistic Boolean networks without perturbations where the underlying Markov chain contains more than one ergodic set, considering the ergodic sets rather than the attractors may be more significant for understanding the long-run behaviour of the network. For example, in the context of modelling biological processes with PBNs, cellular phenotypes may in fact be represented by the ergodic sets. For more details see [32,43,44].

A number of other issues related to probabilistic Boolean network dynamics have been considered in the literature. We briefly list them here. In [45,46], the ordering of network switching and state transitions in context-sensitive PBNs are considered and its influence on the steady-state probability distributions is investigated. Algorithms for enumeration of attractors in probabilistic Boolean networks are discussed in [47]. Stability and stabilisation issues of PBNs are covered in [48]. Further, network transformations from one to another without losing some crucial properties, e.g., the steady-state probability distribution, are considered in [49]. For this purpose the concepts of homomorphisms and *ε*-homomorphisms for probabilistic regulatory networks, in particular PBNs, are developed.

## Construction and inference of PBNs as models of gene regulatory networks

**Figure 5:**
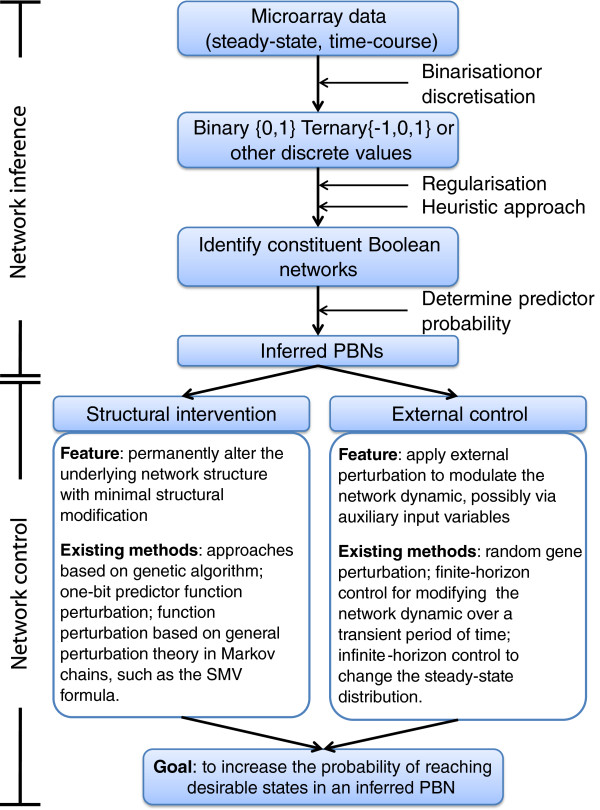
**An outline of the workflow in network inference and control in the PBN framework.** Microarray data, either from steady-state or time-course measurements, are typically binarised or discretised into discrete values. A heuristic approach, such as using genetic algorithms, is generally applied to identify constituent Boolean networks of the inferred PBN. Regularisation methods can be further applied to improve the accuracy of the inference with use of prior information on the network structure or dynamical rules. A number of well-established methods are subsequently applied to determine the predictor probability of each constituent Boolean network, thus the PBN is inferred. The inferred PBN can subsequently be perturbed with the methods on structural intervention or external control. The goal of network control is to increase the probability of reaching desirable states in the corresponding PBN.

One approach to the dynamical modelling of gene regulation is based on the construction and analysis of network models. Generally, in the study of dynamical systems, long-run behaviour characteristics are of utter importance and their determination is a main aspect of system analysis. Reversely, the task of constructing a network possessing a specific set of properties is a subject of system synthesis. However, this *inverse problem* is usually ill-posed, i.e., there may be many models, or none, with the given properties [50]. Here we concentrate on the problem of inference from data in the framework of probabilistic Boolean networks, an inverse problem in which a network is constructed relative to some relationship with the available data. An outline of the workflow in network inference in the PBN framework is shown in Figure 5.

A data-driven approach for model construction consists of inferring the model structure and model parameters from measurement data, which in the case of gene regulation most commonly are gene expression measurements obtained with microarray technology. However, such data are continuous in nature. Thus, prior to the inference of Boolean or other discrete-type models (e.g., ternary) the measurements are usually discretised. The most common discretisation is binary (0 or 1) or ternary(usually -1, 0, 1) [8]. Discretisation is often justified as biological systems commonly exhibit switch-like on/off behaviour. Moreover, there are also a number of pragmatic reasons for quantising the measurements, e.g., it reduces the level of model complexity implying less computation and lower data requirements for model identification, provides a certain level of robustness to noise in the data, and has been shown to substantially reduce error rates in microarray-based classification [8,51-53]. A number of methods for discretisation of gene expression data exist, many of them having their origin in signal processing. One approach to quantisation was proposed in [54]: given some thresholds *τ*_1_ < *τ*_2_ < … (e.g., corresponding to limiting cases of a sigmoidal response), a multilevel discrete variable **x** is defined as **x** = *φ*(*x*) = *r*_*k*_ for *τ*_*k*_ < *x* ≤ *τ*_*k*+1_. As mentioned in [8], the thresholds can either come from prior knowledge or be chosen automatically from the data. In fact, there are various ways for optimal selection of the thresholds *τ*_*k*_. One of the most popular methods is the Lloyd-Max quantizer, which amounts to minimising a so-called mean square quantisation error, see [55] for details. Approaches specific to binarising gene expression data can be found in [56-58]. Recently, Hopfensitz et al. [58] proposed a new approach to binarisation which incorporates measurements at multiple resolutions. The method, called Binarization across Multiple Scales, is based on the computation of a series of step functions, detection of the strongest discontinuity in each step function and the estimation of the location and variation of the strongest discontinuities. Two variants of the method are proposed which differ in the approach towards the calculation of the series of step functions. The proposed method allows thresholds determination even with limited number of samples and simultaneously provides a measure of threshold validity – the latter can further be used to restrict network inference only to measurements yielding relevant thresholds. An example of application of binarisation to real data in the context of modelling with PBNs can be found in [10], where a brain connectivity network of Parkinson’s disease is analysed. Binarisation is performed on fMRI real-valued data along the method recently proposed in [59].

One of the most straightforward inferential approaches is the *consistency problem* (also referred to as the *extension problem*), that entails a search for a rule from experimental data [8,60-62]. The problem amounts to finding in a specified class of Boolean functions one that complies with two given sets of “true" and “false" Boolean vectors, i.e., a function that takes the value 1 for each of the “true" vectors and 0 for each of the “false" vectors.

In the case of real experimental data, a consistent extension may not exist either due to measurement noise or due to some underlying latent factors or other external influences not considered in the model [8]. In such case instead of searching for a consistent extension a Boolean function that minimises the number of misclassifications (errors) is considered. This problem is known as the *best-fit extension problem*[61] and is computationally more difficult than the consistency problem, since the latter is a special case of the former.

The application of PBN for modelling of large-scale networks is often impeded by limited sample sizes of experimental data. As mentioned in [63], main challenges in automated network reconstruction arise from the exponential growth of possible model topologies for increasing network size, the high level of variability in measured data often characterised by low signal to noise ratios, and the usually large number of different components that are measured versus relatively small number of different observations under changing conditions, e.g., number of time points or perturbations of the biological system. Together these problems lead to non-identifiability and over-fitting of models [63]. In such cases any prior information on the network structure or dynamical rules is likely to improve the accuracy of the inference [8,64]. This information usually pertains to model complexity and is used to penalise excessively complex models. For this purpose, the so-called *regularisation methods* can be employed. The most popular regularisation assumption in gene regulatory modelling is that the inferred models should be sparse, i.e., the number of regulators acting on a gene is low [65-68] or that the node degree in biological networks is often power law distributed, with only few highly-connected genes, and most genes having small number of interaction partners [63,69]. Regularisation is a well-established inference approach in the framework of Bayesian networks (see, e.g., [63,70,71]) and can be also used in the framework of BNs and PBNs. For example, in the case of inference of Boolean networks, the so-called *sensitivity regularisation method* has been proposed [64]. Due to limited sets of data, the estimates of the errors of a given model in the best-fit extension problem, which themselves depend on the measurements, may be highly variable [64]. The regularisation is built on the observation that the expectation of the state transition error generally depends on a number of terms, among others the sensitivity deviation which is a difference in the sensitivities of the original and the inferred networks. In consequence, as argued in [64], the sensitivity deviation can be incorporated as an additional penalty term to the best-fit objective function, reflecting the hypothesis that the best inference should have a small error in both state transition and sensitivity.

In order to infer a PBN, strong candidates for regular Boolean networks need to be identified first. This can be performed with generic methods mentioned in [72] such as literature data compilation, the gene association networks approach [73,74] or by applying a heuristic approach, e.g., a genetic algorithm, which searches through the model space to find good candidates for the network structure with respect to a specified fitness function. Next, the candidates’ predictor functions are combined into a set of network transition functions for the PBN. An example of PBN model selection using heuristics can be found in [75].

A common strategy for determining the predictor probabilities relies on the *coefficient of determination* (CoD) between target and predictor genes [8,32,72,76]. The CoD is a measure of relative decrease in error from estimating transcriptional levels of a target gene via the levels of its predictor genes rather than the best possible prediction in the absence of predictor genes [8]. The CoDs can be then translated to the predictor probabilities. However, as pointed out in [77], for each gene, the maximum number of possible predictors as well as the number of their corresponding probabilities is equal to $2^{2^{n}}$, where *n* is the number of nodes. This implies that the number of parameters in the PBN model is $O(n2^{2^{n}})$^e^. Therefore, the applicability of the CoD approach is significantly limited due to the model complexity or imprecisions owing to insufficient data sample size. This hindrance is often surpassed by imposing some constraints on the maximum size of admissible predictors for each gene.

In [50] the authors consider the *attractor inverse problem*, that involves designing Boolean networks given attractor and connectivity information. Two algorithms for solving this problem are proposed. They are based on two assumptions on the biological reality: first, the biological stability, i.e., that most of the steady-state probability mass is concentrated in the attractors and, second, the biological tendency to stably occupy a given state, i.e., attractors are singleton attractor cycles consisting of a single state. The first algorithm operates directly on the truth table, while taking into account simultaneously the information on the attractors and predictor sets. There is however no control on the level-set structure. The second algorithm works on the state transition diagram that satisfies the design requirements on attractor and level-set structures and checks whether the associated truth table has predictor sets that agree with the design goals. The proposed algorithms can be further used in a procedure for designing PBN from data. In the approach described in [50], a collection of BNs is generated by the first algorithm, then some of the BNs are selected based on the basin sizes criterion and combined in a PBN whose steady-state distribution closely matches the observed data frequency distribution. This design procedure has been applied to gene-expression profiles in a study of 31 malignant melanoma samples in [50].

An inverse PBN construction approach is also described in [78]. This work relies on expressing the probability transition matrix as a weighted sum of Boolean network matrices. A heuristic algorithm with *O*(*m*2^*n*^) complexity is proposed, where *n*, *m* stand for the number of genes, respectively the number of non-zero entries in the transition matrix. The authors also introduce an entropy based probabilistic extension, both algorithms being analysed against random transition matrices.

Usually, the optimal predictor for a gene will not be perfect as there will be inconsistencies in the data. In [79] it is proposed to model these inconsistencies in a way that mimics context changes in genomic regulation, with the intention to view data inconsistencies as caused by latent variables. The inference procedure of [79] results in PBNs whose contexts model the data in such a way that they are consistent within each context. The key criterion for network design is that the distribution of data states agrees with the distribution of expected long-term state observations for the system.

The probabilities of the system being in a particular context and the number of constituent networks are determined by the data. The approach of [79] can be seen as imposing a structure on a probabilistic Boolean network that resolves inconsistencies in the data arising from mixing of data from several contexts. It should be noted that in this approach the contexts are determined directly by the data, whereas in [32] and [80] constituent networks depend on the number of high-CoD predictor sets or high Bayes-score predictor sets, respectively, and these in turn depend on the designer’s choice of a threshold. Moreover, the number of constituent networks is determined by how inconsistencies appear in the data, not the number of states appearing in the data (see [8] for an example). The contextual-design method of [79] has been applied to expression profiles for melanoma genetic network.

We just mention here that also information theoretic approaches were considered for inference of PBN from data. Probably the most widely studied methods are based on the minimum description length (MDL) principle [81]. Descriptions of inference algorithms that utilise this principle can be found, e.g., in [8,82,83].

The manner of inference depends on the kind of experimental data available. There are two cases: 1) time-series data and 2) steady-state data. We proceed with presenting them briefly.

### Time-course measurements

It is assumed that the available data are a single temporal sequence of network states. In this case, given a sufficiently long sequence of observations, the goal is to infer a PBN that is one of plausible candidates to have generated the data. Usually, an inference procedure for this type of problem constructs a network that is to some extent consistent with the observed sequence.

In [84,85], the inference in case of context-sensitive PBNs with perturbations is considered, where the probability of switching from the current constituent Boolean network to a different one is assumed to be small. The proposed inference procedure consists of three main steps: first, identification of subsequences in the temporal data sequence that correspond to constituent Boolean networks with use of so-called ‘purity functions’; second, determination of *essential predictors* for each subsequence by applying an inference procedure based on the transition counting matrix and a proposed cost function; finally, inference of perturbation, switching, and selection probabilities. However, the amount of temporal data needed for inference with this approach is huge, especially due to the perturbation and switching probabilities: if they are very small, then long periods of time are needed to escape attractors and if they are large, estimation accuracy is harmed. As stated in [85], if one does not wish to infer the perturbation, switching, and selection probabilities, then constituent-network connectivity can be discovered with decent accuracy for relatively small time-course sequences.

A more practical way of inferring PBN parameters from time-course measurements is presented in [77]. The authors propose a multivariate Markov chain model to infer the genetic network, develop techniques for estimating the model parameters and provide an efficient method of estimating PBN parameters from their multivariate Markov chain model. The proposed technique has been tested with synthetic data as well as applied to gene expression data of yeast.

Further, in [86] the problem of PBN context estimation from time-course data is considered. The inference is considered with respect to minimising both the conditional and unconditional mean-square error (MSE). The author proposes a novel state-space signal model for discrete-time Boolean dynamical systems, which includes as special cases distinct Boolean models, one of them being the PBN model. A Boolean Kalman Filter algorithm is employed to provide the optimal PBN context switching inference procedure in accordance to minimisation of MSE.

### Steady-state data

Here we consider a long-run inverse problem in the context of probabilistic Boolean networks as models for gene regulation. On one hand, in the case of microarray-based gene-expression studies it is often assumed that the data are obtained by sampling from a steady state. On the other hand, attractors represent the essential long-run behaviour of the modelled system [31]. Thus, in the modelling framework of Boolean networks it is expected that the observed data states are mostly the attractor states of a model network. In consequence, much of the steady-state distribution mass of the model network should lie in the states observed in the sample data [50,80,87]. In the case of Boolean networks with perturbations or probabilistic Boolean networks with perturbations, the underlying dynamical system is an ergodic Markov chain, hence possesses a steady-state distribution. However, by imposing some mild stability constraints that reflect biological state stability, also in these frameworks most of the steady-state probability mass is carried by the attractors [31].

There are however inherent limitations to the construction of dynamical systems from steady-state data. Although the steady-state behaviour restricts the network dynamics, it does not determine the steady-state behaviour: there may be a collection of compatible networks with a given attractor structure. In particular, it does not determine the Boolean network’s basin structure. As a consequence, obtaining good inference relative to the attractor structure does not necessary entail valid inference with respect to the steady-state distribution as the steady-state probabilities of attractor states depend on the basin structure [50,80]. In fact building a dynamical model from steady-state data is a kind of over-fitting [88].

Although the CoD has been used for inference of PBNs from steady-state data in [32], a fundamental problem is that the CoD cannot provide information on the direction of prediction without time-course data. The resulting *bidirectional relationships* can affect the inferred graph topology by introducing spurious connections. Moreover, they can lead to inference of spurious attractor cycles that do not correspond to any biological state [8]. As a consequence, this suppressed the use of the CoD as a inference method for steady-state data.

The inference methods that replaced the CoD approach are primarily based on the attractor structure [50,79] or graph topology [89]. In the former case, the key concern is to infer an attractor structure close to that of the true network. In the latter case, the focus is on the agreement between graph connections, e.g., as measured by the Hamming distance between the regulatory graphs [8]. In [16], an approach that achieves both preservation of attractor structure and connectivity based on strong gene prediction has been proposed.

Another approach to the problem of constructing gene regulatory networks from expression data using the PBNs framework is proposed in [90]. The key element of this method is a non-linear regression technique based on reversible-jump Markov chain Monte Carlo (MCMC) annealing for predictor design. The network construction algorithm consists of the following stages. First, for each target gene *x*_*i*_ (*i* = 1,2,…,*n*) in the network of *n* genes a collection of predictor sets is determined by applying a clustering technique based on mutual information minimisation. Optimisation ^f^ is performed with use of the simulated annealing procedure. This step reduces the class of different predictor functions available for each target gene. Next, each predictor set is used to model a predictor function $f_{k}^{\left(i\right)}$ by a perceptron consisting of both a linear and a nonlinear term, where *k* = 1,2,…,*l*(*i*), with *l*(*i*) the number of predictor sets found in the previous step for target gene *x*_*i*_. A reversible MCMC technique is used to calculate the model order and the parameters. Finally, the CoD is used to compute the probability of selecting different predictors for each gene. For a detailed description of this algorithm and its application to data on transcription levels in the context of investigating responsiveness to genotoxic stresses see [90]. It should be noticed that the proposed reversible-jump MCMC model for predictor design extends the binary nature of PBNs allowing for a more general model containing non-Boolean predictor functions that operate on variables with any finite number of possible discrete values [72].

As an alternative to the technique of [90], a fully Bayesian approach (without the use of CoD) for constructing probabilistic gene regulatory networks, with an emphasis for network topology, is proposed in [80]. In this approach, the predictor sets of each target gene are computed, the corresponding predictors are determined, and the associated probabilities, based on the nonlinear perceptron model of [90], are calculated by relying on a reversible jump MCMC. Then, a MCMC method is used to search for the network configurations that maximise the Bayesian scores to construct the network. As stated in [8], this method produces models whose steady-state distribution contains attractors that are either identical or very similar to the states observed in the data. Moreover, many of the attractors are singleton attractors, which reflect the biological propensity to stably occupy a given state. The approach of [90] has been applied to gene-expression profiles resulting from the study of 31 malignant melanoma samples presented in [91].

In [92] the inverse problem of constructing instantaneously random PBNs from a given stationary distribution and a set of given Boolean networks is considered. Due to large size of this problem, it is formulated in terms of constrained least squares and a heuristic method based on Conjugate Gradient is proposed as a solution.

In [93], the inverse problem of PBNs with perturbations is considered, where a modified Newton method is proposed for computing the perturbation probability *p* where the transition probability matrix $\tilde{A}$ and the steady-state probability of the PBNp $\tilde{x}$ are known. The new algorithm makes use of certain properties of the set of steady-state nonlinear equations, i.e., $\tilde{A}\tilde{x}-\tilde{x}=0$, with *p* as the unknown variable. Considering these properties improves the computational efficiency with respect to a direct approach in which every of the 2^*n*^ equations (*n* being the number of nodes) is solved and common solutions are reported.

## Structural intervention and control of PBNs

Using PBNs for the modelling and analysis of biological systems can lead to a deeper understanding of the dynamics and behaviour of these systems (see Section ‘Dynamics of PBNs’), paving the way for different methods used for system structure inference and data measurement (see Section ‘Construction and inference of PBNs as models of gene regulatory networks’). Another major objective of such studies is to predict the effect a perturbation or an intervention has on the system structure, e.g., allowing to identify potential targets for therapeutic intervention in diseases such as cancer. Intervention strategies in PBNs, e.g., as to change the long-run behavior of networks in order to decrease the probability of entering some undesired state, rely on two different kinds of direction – *structural intervention*[8,33] and *external control*[8,18]. While the first approach can alter the underlying network structure permanently, the second one uses external control to modulate the network dynamics. A classification of network control methods in the PBN framework is shown in Figure 5.

### Structural intervention

The problem of performing a structural intervention in a PBN looks at how the steady-state probability of certain states can be changed with only minimal structural modifications [8,33]. A more formal description is offered in the following. Given a PBN and two subsets *A* and *B* of its states, the associated steady-state probabilities *π*(*A*), *π*(*B*), have to be modified such as to approach some given values *λ*_*A*_, respectively *λ*_*B*_. This can be achieved by replacing the predictor function *f*_*ik*_ (of gene *i* in context *k*) with a new function *g*_*ik*_, while keeping all other network parameters unchanged. We denote the steady-state distribution of the resulting PBN as *μ*. Then, it is possible to interpret the problem as an optimisation one: given the state sets *A*, *B*, and two values *λ*_*A*_ ≥ 0, *λ*_*B*_ ≥ 0, with *λ*_*A*_+*λ*_*B*_ ≤ 1, find a context *k*, a gene *i*, and a function *g*_*ik*_ to replace *f*_*ik*_, such as to minimises *ε*(*A*,*B*) = ∣*μ*(*A*)−*λ*_*A*_∣+∣*μ*(*B*)−*λ*_*B*_∣, with respect to all contexts, genes, and predictor functions. Note that *A* and *B* can be used to represent both desirable as well as undesirable states. While this approach allows changing one predictor function at a time, a generalisation can be made by allowing a number of predictor functions or by adding more constraints on the selected functions, only to give a few examples.

Shmulevich et al. [33] proposed using genetic algorithms to deal with the above optimisation problem. Later, Xiao and Dougherty [94] provided a constructive algorithm for structural intervention and applied it to a WNT5A network. The proposed algorithm focuses on the impact one-bit predictor function perturbations have on state transitions and attractors. Their approach, however, does not directly characterise the steady-state distribution changes that result from (structural) perturbations of a given probability. In order to solve this problem, Qian and Dougherty [95] derived a formal characterisation of optimal structural intervention, based on the general perturbation theory in finite Markov chains. Specifically, they gave an analytical solution for computing the perturbed steady-state distribution by looking at function perturbations. Their work mainly focused on one-bit function (or rank-1 matrix) perturbations, implying that for more general perturbations, one needs to consider an iterative approach. The associated complexity of such an approach is of *O*(2^3*n*^), where *n* is the number of genes in the network. Their results have been applied to a WNT5A network and a mammalian cell cycle related network, respectively. More recently, Qian et al. [96] extended their previous result in [95] to a more efficient solution that uses the Sherman-Morrison-Woodury (SMW) formula [97] to deal with rank-*k* matrix perturbations. Thus, they managed to reduce the computational complexity of the approach from *O*(2^3*n*^) to *O*(*k*^3^), where *k* ≪ 2^*n*^ (*k* is much smaller than 2^*n*^). The application of the derived structural intervention method to a mutated mammalian cell cycle network shows that the intervention strategy can identify the main targets to stop uncontrolled cell growth in the network.

Qian and Dougherty [98] also looked at how long-run sensitivity analysis can be used in PBNs, in terms of difference between steady-state distributions before and after perturbation, and with respect to different elements of the network, e.g., probabilistic parameters, regulatory functions, etc.

### External control

While structural intervention focuses on a permanent change in the network dynamics, *external control* relies on Markov decision processes theory for driving a network out of an undesired state, i.e. as to reach a more desirable one [8,18].

The first approach to deal with PBNs was proposed by Shmulevich et al. [18]. They studied the impact of random *gene perturbations*^g^ on the long-run behavior of a network. The main idea of Shmulevich et al. [18] is to construct a formulation of the state-transition probability that relies on the probability of a gene perturbation and on Boolean functions for finding bounds for the steady-state probability. Their particularly interesting finding is that these states (which in terms of mean first-passage times (MFPT) are easy to reach from other states) are more stable with respect to random gene perturbations. In gene regulatory networks, it is important to identify what genes are more likely to lead the network into a desirable state when perturbed. MFPT naturally captures this idea – a few other methods developed by Shmulevich et al. [18] work, for example, by maximising the probability to enter some particular state in some fixed maximum amount of time, or by minimising the time needed to reach that state.

Gene perturbation works by single flips of a gene’s state, providing a natural platform for external intervention control via auxiliary input variables. It makes sense from a biological perspective, for example, to model auxiliary treatments in cancer such as radiation. The value of these variables can be thus chosen such as to make the probabilistic distribution vector of the PBN evolve in some desired manner.

More formally, given a PBN with *n* genes and *k* control inputs, *u*_1_,*u*_2_,…,*u*_*k*_, the vector *u*(*t*) = (*u*_1_(*t*),*u*_2_(*t*),…,*u*_*k*_(*t*)) is used to denote the values of all control inputs at a given time step *t*. Let *P* denote the transition probability matrix of the PBN, evolving according to *w*(*t*+1) = *w*(*t*)·*P*(*u*(*t*)). It is obvious to see that, at each time step *t*, *P* depends not only on the initial probability distribution vector, but also on the values of the control inputs. External control is essentially about making the network evolve in some desired manner by choosing, at each time step, input control values. The sequence of control inputs, referred to as a *control policy* or *strategy*, can be associated to a cost function which has to be minimised over the entire class of allowed policies. Such functions capture the cost and benefit of using interventions, and are normally application dependent. For the sake of simplicity, we use *J*_*ω*_(*z*(0)) to denote the cost with respect to a control policy *ω* and an initial state *z*(0). Then, an *optimal PBN control problem* can be defined as a search for a control policy *ω* that minimises the cost *J*_*ω*_(*z*(0)). External control in PBNs can be classified into the following two groups.

#### Finite-horizon external control

The *finite-horizon external control* problem is about modifying over a transient period of time the network dynamics of some given PBN, without changing its steady-state distribution. In other words, external control is only applied over a finite number of *M* time steps, using policies of the form *ω* = (*μ*_0_,*μ*_1_,…,*μ*_*M*−1_). The first optimal finite control formulation in PBNs, and a solution based on Dynamic Programming [99], were given by Datta et al. [100]. Working assumptions implied known transition probabilities and horizon length, later removed in [101] by making use of measurements, thought to be related to the underlying Markov chain states of the PBN. Pal et al. [17] extended the results of Datta et al. [100,101] to context-sensitive PBNs with perturbation. The results have been used to devise a control strategy that reduces the WNT5A gene’s action in affecting biological regulation.

Optimal finite-horizon dynamic programming based control, assuming a fixed number of time steps *M* and a fixed number of controls *k*, has a computational complexity of $O(2^{2^{n}})$, where *n* is the number of genes in the network. Namely, the problem is limited by the size of the network as one needs to compute the transition probability matrix. In particular, Akutsu et al. [102] proved that the problem is NP-hard.^h^ Chen and Ching [103] used dynamic programming in conjunction with state reduction techniques [104,105] to find an optimal control policy for large PBNs. They managed to reduce the computation complexity to *O*(∣ *R* ∣), where ∣ *R* ∣ is the number of states after state reduction.

Kobayashi and Hiraishi [106] proposed an integer programming based approach that avoids computing the probability matrix in optimal finite-horizon control. Later, they extended their work to context-sensitive PBNs [107,108], focusing on the lower and upper bounds of the cost function. Furthermore, Kobayashi and Hiraishi [109] proposed a polynomial optimisation approach where a PBN is first transformed into a polynomial system, subsequently allowing to reduce the optimal control to a polynomial optimisation problem. In the above papers, only small examples are used to illustrate the proposed approaches.

Ching et al. [110] looked at hard constraints for an upper bound on the number of controls, and proposed a novel approach that requires minimising the distance between terminal and desirable states. They also gave a method to reduce the computational cost of the problem by using an approximation technique [12]. Cong et al. [111] made one step further by considering the case of multiple hard constraints, i.e., the maximum numbers of times each control method can be applied, developing an algorithm capable of finding all optimal control policies. A heuristic approach was developed by the same authors in order to deal with large size networks [111]. A different and more efficient algorithm, using integer linear programming with hard constraints, was presented later by Chen et al. [112]. The WNT5A network is a typical example used in [111,112].

Instead of minimising the cost, Liu et al. [113] investigated the problem of how control can be used to reach desirable network states, with maximal probability and within a certain time. Later, Liu [19] imposed another new criterion for the optimal design of PBN control policies, namely the expected average time required to transform undesired states into desirable ones. In both papers, the optimal control problem can be solved by minimising the MFPT of discrete-time Markov decision processes.

The controllability problem of PBNs was studied by Li and Sun [114]. A semi-tensor product of matrices, as described in their work, allows to convert a probabilistic Boolean control network into a discrete time system. They provided some conditions for the controllability of PBNs via either open or closed loop control.

#### Infinite-horizon external control

*Infinite-horizon external control* implies working with external auxiliary variables, over an infinite period of time, the steady-state distribution being also changed. Policies in this case have the form of *ω* = (*μ*_0_,*μ*_1_,…).

In the finite-horizon case, the optimal control policy is calculated by (essentially) using a backward dynamic programming algorithm, ending once the initial state is reached. However, this approach cannot be applied to infinite-horizon control directly due to the non-existence of a termination state in the finite-horizon case, potentially leading to an infinite total cost. Pal et al. [115] extended the earlier finite-horizon results to the infinite-horizon case for context-sensitive PBNs. They solved the above two problems by using the theory of average expected costs and expected discounted cost criteria in Markov decision processes. For applications, they considered a gene network containing the genes WNT5A, pirin, S100P, RET1, MART1, HADHB, and STC2.

A robust control policy can be found in Pal et al. [116], devised via a minimisation of the worst-case cost over the uncertainty set, with uncertainty defined with respect to the entries of the transition probability matrix.

Due to the computational complexity of $O(2^{2^{n}})$, several greedy algorithms have been proposed in the literature. Vahedi et al. [117] developed a greedy control policy that uses MFPT. Their main idea is to reduce the risk of entering undesirable states by increasing (or decreasing) the time needed to enter such a state (or, respectively a desirable state). Performance of the MFPT-based algorithm was studied on a few synthetic PBNs and a PBN obtained from a melanoma gene-expression dataset, where the abundance of messenger RNA for the gene WNT5A was found to be highly discriminating between cells with properties associated with high or low metastatic competence. Later, three different greedy control policies were proposed by Qian et al. [118], using the steady-state probability mass. The first one explores the structural information of a basin of attractors in order to reduce the steady-state probability mass for undesirable states, while the remaining two policies regard the shift in the steady-state probability mass of undesirable states as a criterion when applying control. The identified three policies, together with the one based on MFPT [117], were evaluated on a large number (around 1000) of randomly generated networks and a mammalian cell cycle network [119].

Some types of cancer therapies like chemotherapy, are given in cycles with each treatment being followed by a recovery period. Vahedi et al. [120] showed how an optimal cyclic control policy can be devised for PBNs. Yousefi et al. [121] extended the results in [120] to obtain optimal control policies for the class of cyclic therapeutic methods where interventions have a fixed-length duration of effectiveness. Both of the two approaches [120,121] were applied to derive optimal cyclic policies to control the behavior of regulatory models of the mammalian cell cycle network [119]. While the goal of control policies is to reduce the steady-state probability mass of undesirable states, in practice it is also important to limit collateral damage, to consider when designing control policies. Based on this observation, Qian and Dougherty [122] developed two new phenotypically-constrained control policies by investigating their effects on the long-run behaviour of the network. The newly proposed policies were examined on a reduced network of 10 nodes. The network was obtained from gene expression data collected for the study of metastatic melanoma (e.g, see [91]).

## Relationship between PBNs and other probabilistic graphical models

Probabilistic graphical models, commonly applied in computational biology for network reconstruction, provide the means for representing complex joint distributions. Examples include PBNs, Bayesian networks and their variants, e.g., dynamic and hierarchical Bayesian networks, hidden Markov models, factor graphs, Markov random fields, conditional random fields, Markov logic networks, etc. In this section we discuss the relationship between the two of them which are usually employed to deal with system dynamics: the PBNs and the dynamic Bayesian networks, the latter generalising hidden Markov models.

A Bayesian network is essentially a graphical, compact representation of a joint probability distribution. The Bayesian network consists of two elements. First, a directed acyclic graph (DAG) where the vertices of the graph represent random variables and the directed edges or lack thereof encodes the so-called *Markovian assumption*, which states that each variable is independent of its non-descendants, given its parents [8,123]. Second, a set of local conditional probability distributions for each vertex, given its parents in the graph. By the chain rule of probabilities, the joint probability distribution on the random variables in the graph can be decomposed into a product of the local conditional probabilities, i.e., if there are *n* random variables *x*_*i*_, *i* = 1,2,…,*n* and Pa(*X*_*i*_) denotes the parents of *x*_*i*_ in the graph, then the joint probability distribution factors as

(8) $$\text{Pr}(X_{1},X_{2},\ldots ,X_{n})=\overset{n}{\underset{i=1}{\prod }}\text{Pr}(X_{i}|\text{Pa}(X_{i})).$$

Two different Bayesian networks can encode the same set of independencies. Such networks are said to be *equivalent*. Equivalent networks cannot be distinguished when inferring the network from measurement data. One way to bypass this difficulty is to perform targeted intervention experiments which can narrow the range of possible network architectures.

Dynamic Bayesian networks (DBNs) are extensions of Bayesian networks to the temporal domain and can be used to model stochastic processes [70]. DBNs generalise hidden Markov models and linear dynamical systems by representing the conditional dependencies and independencies between variables over time. Contrary to Bayesian networks, DBNs can be used to model feedback relationships, a ubiquitous element in genetic regulation. In comparison to PBNs, dynamic Bayesian networks support the assignment of quantitative state values, making this modelling approach more flexible to handle various types of data. DBNs are broadly applied to represent biological networks such as gene regulatory networks [124-127], signal transduction networks, e.g., [128-130], metabolic networks [131], as well as networks in physiology and medicine [132-136].

As shown in [137], PBNs and binary-valued DBNs whose initial and transition Bayesian networks are assumed to have only within and between consecutive slice connections, respectively, can represent the same joint probability distribution over their common variables. This is true both for independent as well as dependent variants of PBNs. However, there are many statistically equivalent PBNs that correspond to a DBN. On one hand, the PBN framework can be considered as redundant from the probabilistic point of view. On the other hand, it is richer from the functional point of view because it models the regulatory roles of different gene sets in more detail than the conditional probabilities in DBNs [137]. The conversion algorithms between the two modelling formalism are presented in [137], both for independent and dependent PBNs. Also the extensions of standard PBNs to context-sensitive PBNp is discussed. The perturbations and context switching can be introduced in the DBN formalism by adding additional hidden nodes to the dynamic Bayesian network, as shown in [137].

In terms of applications, it has been shown that both the PBN and the DBN approaches principally have good performance on the inference of gene regulatory networks from microarray data [138]. In addition, the connection between PBNs and DBNs makes it possible to apply the advanced DBNs to PBNs tools and vice versa. For example, an abundant collection of learning theory and algorithms for DBNs already exists and methods for the analysis of temporal behaviour of DBNs are already established. These techniques can be tailored to be applied directly in the context of PBNs. Conversely, the tool for controlling the steady-state behaviour of the networks, tools for network projection, node adjunction, resolution reduction as well as efficient learning schemes can be applied to DBNs.

As presented in [139], PBNs and dynamic Bayesian networks can be viewed as consisting of a probabilistic (Markov chain) and of a (Boolean) logic component. In the case of a dynamic Bayesian network, the probabilistic component is defined by a conditional probability chain rule and a Markov chain while the logic component is given by propositional logic with structural requirements. As shown in [139], Bayesian networks, with their hierarchical and dynamic variants, as well as probabilistic Boolean networks, are all generalised by Markov logic networks. The same separation of components applies. For a Markov logic network, the probabilistic component is a Markov random field and the logic component is the first order logic. We refer to [139] for more details on this framework, its applications in biology and medicine as well as the relationship with Bayesian networks.

## PBN applications in biological and biomedical studies

### PBN models for the representation of biological networks

Even though a significant part of the research on PBNs is theoretical, a large number of applied studies on the use of PBNs for various biological systems can be found in the literature. This is particularly the case with inference of models for molecular and physiological networks (from prior knowledge or data), with subsequent model analysis that leads to novel knowledge in biology and medicine.

#### PBNs as models of gene regulatory networks

PBNs were originally developed as models for Gene Regulatory Networks (GRNs) [3,8]. As stated in [32], PBNs 1) incorporate rule-based dependencies between genes; 2) allow the systematic study of global network dynamics; 3) are able to cope with uncertainty, both in the data and model selection; and 4) permit the quantification of the relative influence and sensitivity of genes in their interactions with other genes. In the PBN modelling framework, gene expression is quantised to two levels: ON and OFF.

The dynamical behaviour of PBNs can be used to model many biologically meaningful phenomena, such as cellular state dynamics possessing switch-like behaviour, hysteresis, stability, and etc. [32,140]. Often, the attractor cycles are interpreted as functional states on physiological time scales or as cellular phenotypes on developmental time-scales [7,8]. This interpretation is fairly reasonable as most cell types are characterised by stable recurrent patterns of gene expression [31].

In the past years, there were several studies which successfully applied PBNs for the construction of GRNs from high-throughput gene expression microarray experiment data. In 2006, Yu et al. inferred a GRN of the interferon pathway in macrophages using time-course gene expression data [22]. The optimal network was identified applying the CoD approach. It was shown that the respective selection probabilities are varying for different biological conditions, e.g., after interferon treatment or after viral infection on macrophage, while the structure of the constituent network, i.e., predictor functions, remains stable. With a similar approach, Nguyen et al. inferred a GRN of hepatocellular carcinoma from microarray data and compared it to a network derived from control non-cancerous samples [141]. They indicated that certain genes in tumour samples show activity in steady-state periods while there is no activity for these genes in the control (non-cancerous) samples. This allowed to distinguish different gene regulatory processes being realized with the same set of genes.

Hashimoto et al. modelled the cell cycle of budding yeast by using context-sensitive PBNs [23]. They showed that the switching behaviour from stationary G1 phase to excited G1 phase in the PBN model is more frequent, when compared to the stochastic model of Zhang et al. [142]. Recently, Todd et al. identified the ergodic sets of states in PBNs that correspond to each phase of the budding yeast cell cycle, which in turn correspond to the cellular phenotypes [44]. The analysis of the dynamical behaviour gave additional insights on yeast cell cycle regulation, e.g., the yeast cell cycle network showed robustness both to external variable environments and to certain perturbations such as nitrogen deprivation, where yeast cells proceeded through one round of division and arrest at G1 phase without appreciable growth.

In 2011, Flöttmann et al. modelled the regulatory processes that govern the production of induced Pluripotent Stem (iPS) cells by considering the interplay between gene expression, chromatin modification, and DNA methylation [24]. As there is no clear guideline on how to assign Boolean functions to represent the interactions of each gene, their PBN model was designed to work by representing uncertainty via two assignments. First, a number of possible functions were assigned to the corresponding nodes with different probabilities. Second, the influences of certain nodes were split into separated Boolean functions with varied selection probabilities. A flexibility was thus allowed for choosing Boolean functions that fit the experimental data. With their PBN model, an extensive analysis was performed, allowing to demonstrate epigenetic landscape changes from differentiated cells to iPS cells as a function of time step. In addition, by looking at model variants of the core iPS regulation, it was shown that an increased chromatin modification rate could improve reprogramming efficiency while faster changes in DNA methylation could provide an enhanced rate though at the price of trading-off efficiency.

#### PBN within signal transduction network and metabolic network modelling

To date, there is no study which specifically applied PBN as a stand-alone framework for modelling signal transduction or metabolic networks. Nevertheless, PBN was combined with other algorithms or modelling frameworks. Fertig et al. presented *GESSA*, Graphically Extended Stochastic Simulation Algorithm, a mechanistic hybrid model which integrates the network model of cell signalling with pooled PBN to a differential equation-based model of transcription and translation computed by a stochastic simulation algorithm [25]. The cell signalling PBN model is generated by simulating individual protein copies with the corresponding state transitions updated according to the rules in the PBN. The sum of the resulting molecular states across copies, i.e., of each individual species, is compared to the initial state, the difference being afterwards returned and the cellular state being updated. *GESSA* was applied to the study of the cell fate decision of valval precursor cells in *C. elegans*, where model predictions matched the experimental results even for minimal parameterisations of the PBN. It was thus shown that PBN could be an essential component when flexibility is needed in multi-level data integration and model construction.

In metabolic modelling, Chandrasekaran et al. presented an automated algorithm for the Probabilistic Regulation of Metabolism (*PROM*), allowing to reconstruct a probabilistic GRN integrated with a metabolic network from high-throughput data [26]. *PROM* makes use of conditional probabilities to model transcriptional regulation, similar to the CoD concept in PBN inference. This formalism permits the strength of transcription factor (TF)-gene regulation as well as gene states to be represented in terms of probabilities. *PROM* was used to generate a genome-scale integrative transcriptomic and metabolomic network of *Escherichia coli*, where *PROM* surpassed the state-of-the-art methods such as the regulatory flux balance analysis. *PROM* was also used to generate an integrative model of *Mycobacterium tuberculosis*. The results from the model analysis offered additional details on known regulatory mechanisms and also helped to uncover the function of less studied genes on metabolic regulation.

Apart from these two studies, several other works also made use of a probabilistic framework for analysing signal transduction and metabolic networks. Kaderali et al., for instance, developed an algorithm that reconstructs signalling pathways from gene knockdown data (RNAi data) [143]. In this work, pathway topologies are inferred by using Bayesian networks with probabilistic Boolean threshold functions. The algorithm was used to study the Janus Kinase and Signal Transducers and Activators of Transcription (JAK/STAT) pathway, correctly reconstructing the core topology of the pathway along with model variants. Similarly, Sauer et al. [144] used probabilistic equations to determine flux ratios, allowing to express the relative contribution of certain metabolites or pathways as modulators in the network. This assignment is more realistic than using flux absolute integer numbers, given that the flux of each source can relatively contribute to the production of certain metabolites.

#### PBN applications in the context of physiology

PBNs were also used in the recent years for studying networks in physiology, with a close link to medicine. Tay et al. described a dengue hemorrhagic fever (DHF) infection model which contains the interplay between dengue virus and different cytokines which are cross-regulated in T-helper 1 (Th1) and Th2 cells [9]. In their work, a single probabilistic Karnaugh-Map is generated, modelling the inducement probability of each cell as to define the overall influence of inducing nodes. Simulation results matched clinical data for both synchronous and asynchronous updating, with respect to the form and the average duration-based attractors, respectively. In addition, by applying a genetic algorithm [145] to modulate the DHF attractor basins to dengue fever (DF) basins (a less severe form of DHF), Tay et al. also identified the tumour growth factor beta (TGF *β*), interleukine-8 (IL-8) and IL-13, as sensitive intervention points.

Another example in this field can be found in the study of Ma et al., where, based on functional Magnetic Resonance Imaging (fMRI) data, the authors developed a brain connectivity network model for Parkinson’s disease [10]. A method similar to the one of Yu et al. [22] was used for probability inference selection, i.e., the calculation of CoDs. Then the CoDs were subsequently used to generate an influence matrix representation of the brain signal connectivity among brain components. The obtained results showed that a significant difference in connectivity exists for many paired brain-components comparing between normal, Parkinson’s disease with drug, and Parkinson’s disease with drug withdrawal conditions, and this difference was expressed in terms of estimated range of coefficient mean activity. This particular information may allow to construct a new screening procedure for Parkinson’s disease diagnose and to determine drug trial responsiveness based on a non-invasive, fMRI-based investigation in the future.

A certain number of the previously described (applied research) articles on PBN have applications not only in molecular biology, but also in physiology or medicine. Only to name a few examples, being able to distinguish among the regulatory networks of cancer and healthy cells, as presented by Nguyen et al., could contribute to an early detection of cancerous genes in susceptible populations [141]. A better understanding of dynamic processes and the control of somatic cell programming, as proposed by Fertig et al., may lead to a future use of iPS cells in cell or tissue replacement therapies [25]. Last but not least, the *PROM* algorithm, as introduced by Chandrasekaran et al., is capable of predicting transcription factor drug targets which are major hubs in the cellular network of pathogenic organism such as *Mycobacterium tuberculosis*[26]. A further development of drugs in this direction may help in the treatment of different infectious diseases. This new line of treatment could have a strong impact for third-world countries where infectious diseases still remain a major cause of death.

### PBN for Systems Biology and Systems Biomedicine?

As previously discussed, the PBN framework is a topic of intensive and continuous theoretical research with successful applications in the biomedical area. To describe and extend a vision on future PBNs’ applications, we summarise additional arguments to support why this modelling approach is suitable for future research in Systems Biology and Systems Biomedicine.

#### Data integration

Different types of biological and clinical investigation datasets, ranging from qualitative to high-throughput quantitative experimental data, were successfully applied in PBN inference and analysis. Yu et al. [22] and Nyugen et al. [141], for instance, inferred GRNs of macrophages and hepatocellular carcinoma using microarray gene expression data. Flöttmann et al. [24] built a comprehensive epigenetic regulatory network of iPS cells based on gene expression, chromatin modification and DNA methylation data generated from multiple high-throughput experiments. Ma et al. applied voxel selection on fMRI clinical data to capture the activities of each brain’s compartment as the inputs for learning a functional brain connectivity network [10].

We have recently shown that the normalised activity of signalling proteins from quantitative western blot experiment can be compared to the steady-state probability of certain molecule to be ON in instantaneously-random PBNs. In an ergodic model, the activities of signalling proteins, usually given by their phosphorylated forms normalised to the maximal signal, could be correlated with the steady-state probability distribution on the state space of the PBN model. With this regard, PBN could support the integration of semi-quantitative experimental data. Apart from quantitative western blot data, the profiles of signalling proteins from alternative experiments such as enzyme-link immunosorbent assay (ELISA) and high-throughput protein array data are also compatible with this framework (publication submitted).

**Figure 6:**
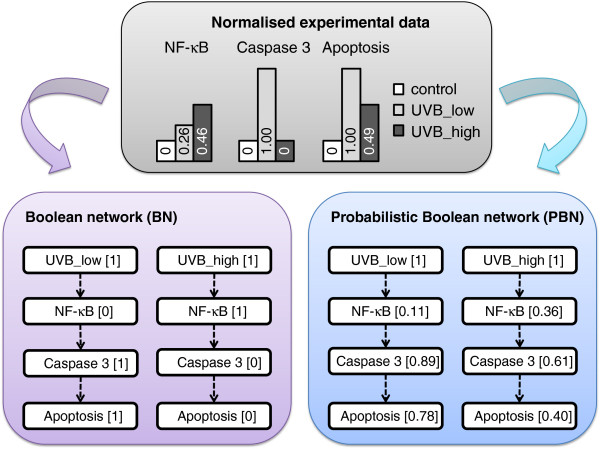
**A comparative study of apoptotic signalling in the context of Boolean and probabilistic Boolean networks.** Background subtracted and normalised experimental data derived from Schlatter et al. is shown in the top box. The experimental data compare the activities of downstream signalling molecules and apoptotic activity in the control setting (no stimulation) versus two intensities of UVB irradiation (UVB_low, 300 *J*/*m*^2^ and UVB_high, 600 *J*/*m*^2^). The activities of caspase 3 refer to the high caspase 3 activities of the original publication. The steady-state values from the original BN and the steady-state probability of the molecules to be ON from the optimised PBN of one exemplifying run are shown in brackets. (Note: The interactions between each node in the actual network are much more complex than the simplified diagram as shown.).

The PBN framework also allows for the description and analysis of large-scale models, for instance as in the case of a Boolean model of apoptosis of Schlatter et al. [146]. Therein, a PBN model was derived from the original literature-based BN consisting of 86 nodes and 125 Boolean interactions. Quantitative experimental data in this study were normalised to the maximal signals across experiments and were used as input data for the PBN model. We analysed the strengths of canonical pathways and crosstalk interactions between different signalling components among apoptotic and related signalling pathways through the identification of selection probability. It was possible to obtain these via optimisation. Thereby a curated signal transduction network topology was derived. The resulting PBN demonstrates the correlation between UVB irradiation, NF *κ*B, caspase 3, and apoptotic activities in a semi-quantitative manner which could not be demonstrated by the original BN. The analysis pointed at an inconsistent caspase 3 measurement, which shows no activity for high UVB irradiation while significant apoptosis is measured (see Figure 6, publication submitted).

Furthermore, the PBN framework has a good potential to describe cellular dynamics at multiple levels. Hybrid PBN-related models could be applied, as previously described, e.g., in the studies of Fertig et al. and Chandrasekaran et al. [25,26]. As reviewed in detail by Gonçalves et al. [147], bridging layers towards an integration of signal transduction, regulation and metabolism into mathematical models still posts many challenges as each of the biological layer has their own distinct characteristics and therefore is suitable for only a subset of modelling approaches. To address such challenges, an integrative hybrid model for flux balance analysis was proposed, combining BN modelling for the gene regulatory part, ODE modelling for the signal transduction part and flux balance analysis for the metabolic part. With this regard, PBN could also be integrated as part of such a hybrid model to describe GRNs and/or signalling networks to provide more details on modelling analysis and interpretation comparing to traditional BNs.

#### Computational tools for PBN modelling and analysis

Several PBN modelling and analysis tools were continuously developed over the past recent years. The *BN/PBN* MATLAB-based toolbox, introduced by Lähdesmäki and Shmulevich in 2003 [27], deals with the simulation, analysis (network statistics, state transitions and distributions), visualisation and intervention analysis of both BN and PBN models. The toolbox was specifically designed for GRN inference and it makes use of CoD calculations. State transition probabilities and influence values (the indicators for interactive effect for each pair of genes) are subsequently calculated based on these calculated CoDs. Ma et al. successfully applied the *BN/PBN* toolbox to infer and analyse the brain connectivity network of Parkinson’s disease patients, as previously described in Section ‘PBN applications in the context of physiology’.

Hinkelmann et al. introduced *ADAM* (Analysis of Discrete Models of biological systems using computer Algebra) [28], a web-based tool for rapid steady-state identification in various discrete model types. The tool automatically converts discrete models into polynomial dynamic systems, allowing to run computer-based algebra analysis. For probabilistic networks, *ADAM* generates a graph of all possible (local rule) updates, thus being capable to build an enumeration of all steady states. *Boolnet*, as introduced by Müssel et al., is an R-package for the generation, modelling, reconstruction and analysis of both synchronous and asynchronous BNs or PBNs [29]. The toolbox features time-series (experimental data) based network inference, e.g., making use of Markov chain simulations for attractor identification with subsequent visualisation and robustness analysis via network perturbation or heuristic search and random walks. We have recently developed *optPBN*, a MATLAB-based toolbox for PBN optimisation based on the *BN/PBN* toolbox. PBNs can easily be constructed from Boolean rule-based models. The toolbox also provides a flexible platform for data integration (e.g., to integrate data from multiple experiments). Different algorithms can be used to address the resulting optimisation problem. Thus, based on normalised protein activity at steady-state data, one can identify a curated model structure from different candidate models. Subsequent analysis on the curated PBN can be performed in the *BN/PBN* toolbox (publication submitted).

We also discuss a few different algorithms and tools which are not specifically designed for PBN but with a high potential for the analysis of PBNs. *PROM*, for example, offers a mean to calculate the flux activities of a metabolic network in a probabilistic manner based on gene expression data [26]. Specifically, this gives rise to the applicability of the PBN framework for metabolic models. Recently, Terfve et al. introduced *CellNOptR*, a flexible toolkit for training protein signalling networks based on a multiple logic formalism [148]. *CellNOptR* offers support for optimisation with respect to multiple modelling frameworks, ranging from logical to ODE (logic rule derived) models. Extending *CellNOptR* towards a probabilistic modelling framework is also foreseen for future work.

#### A perspective on potential applications of PBNs in a clinical setting

It has been a decade since the completion of the Human Genome Project in 2003 that initiated the era of biological and medical investigation in omic scales [149]. Due to technology advancements, the costs of genome sequencing and high throughput biomedical investigations are exponentially decreased and they might become part of the routine medical investigations in a foreseeable time frame [150]. Datasets from omics experiments usually consist of large lists of numbers that represent genes, transcripts, proteins, or metabolites depending on the method applied. In the near future all these methodologies might be applied together routinely, even in time series examinations. The major problem with such data is their high complexity and the need to make them interpretable by the medical staff. Therefore, there is a strong demand for reasonable computational approaches to integrate multidimensional “big data" [151]. In addition, given the rich sets of information from individual patients that physicians will acquire, smart approaches are mandatory to translate and simplify these large-scale biomedical data. Such approaches should facilitate a physician’s decision-making process to provide more accurate diagnosis and optimal treatment.

For these fields we identify the PBN framework as a powerful tool. Recent applications of PBN modelling of gene regulatory and signalling networks have been described in the previous section. As previously summarised, PBNs allow an effective visualisation of GRN models [9,10], allowing to represent gene function and activity [152]. These efforts foster the understanding of gene-gene interactions, consequences of aberrant gene function and targeted perturbations of such networks, as well as finding out the least adverse effects of perturbations [9,153]. PBNs allow for the integration of information from large data sets and for inferring logical relationships between genes/networks. This feature is of particular benefit as many relationships and structural connections among genes are not known. Unknown relationships between transcripts and proteins can also be assessed. In a therapeutic perspective PBNs could be used in a disease-relevant context because many, foremost chronic diseases, share probably common underlying mechanisms that are not elucidated so far [154]. Using PBNs in the study of disease-related networks could enable us to take genetic interactions into account and associations could be generated to identify comorbidities sharing common causative factors. Skahanenko et al. for instance have applied Markov logic networks, a probabilistic logic modelling approach in the same category as PBN, to explore gene-phenotype associations. Whereas traditional statistical methods are employed to identify the marker that associates the most with an observed phenotype, Markov logic networks can be used to identify a subset of markers that predicts the phenotype. Within this method, the relationship between the genetic markers and phenotype(s) can be hypothesised and modelled. All models can then be tested and their respective probability can be derived [139].

In the context of a single, yet complex disease, the study on brain connectivity in Parkinson’s disease by Ma et al. [10] is a good example showing how a probabilistic model such as PBN could translate large-scale biomedical data into a potential application in clinic. fMRI principally measures blood oxygen level-dependent signals that are correlated to the blood flow into different regions of the brain, which in turn give physicians information on the functional activity of specific areas of the brain [155]. For some neurological disorders, such as Parkinson’s disease, the lesions mainly affect a specific area of the brain such as basal ganglia, but have consequences on the overall integrity of brain connectivity, especially on the dopaminergic pathway-dependent motor and cognitive control [156]. Therefore, considering the aetiology and disease progression from only conventional MRI data which demonstrate only structural information is certainly insufficient to yield a comprehensive understanding on the course of disease. Considering diseases as network perturbations [157], the PBN model from Ma et al. demonstrated differences of brain connectivity networks comparing healthy population and diseased cases with and without medication. Such observations could possibly be further developed towards clinical biomarkers which could then be added to physicians’ portfolio and in turn facilitate diagnostic process, treatment design, and follow-up strategy.

Generally, the incorporation of tentativeness and probability could be evolved into a valid concept in a clinical setting, as routine medical investigation often provides no conclusive data. Together with a comprehensive reduction and translation of large-scale and complex biomedical data, the PBN framework might serve as a mean to develop simplistic terms like a probability score for certain condition, e.g., for having a disease or of being responsive to treatment. Such a probabilistic score could serve as a simple but powerful additional input for physicians in order to improve their healthcare management. As a whole, healthcare systems would benefit from reducing costs related to unnecessary diagnostic investigations and treatment failures.

## Conclusion

Even though the concept of PBN for the modelling of biological systems is still young compared to other modelling approaches, a broad area of research activities on this modelling approach such as network inference and network control have been well-established and are continuously developed. For a meaningful comparison of different inference algorithms in the future, it is necessary to quantify their performance. The prospective research in the area of network inference is to develop a formal framework for validation of network inference procedures. Moreover, there is a demand for establishing the properties of network inference procedures under various conditions, e.g., model class, distance function, etc. The current trend in structural intervention and external control is to develop new methods to reduce their computational complexity and to define the optimal control problems and find the corresponding optimal policies for specific therapies. With its flexibility for data integration and the availability of supporting algorithms and computational tools, PBN is one of the most suitable modelling frameworks to describe and analyse complex biological systems from molecular to physiological levels with possible future application at clinical level.

## Endnotes

^a^ In general, *γ*_1_,*γ*_2_,…,*γ*_*n*_ need not be independent and identically distributed random variables, but for the simplicity of presentation are assumed so.

^b^ A state in a Markov chain is said to be ergodic if returns to the state can occur at irregular times and the state is positive recurrent. If all states in an (irreducible) Markov chain are ergodic, then the chain itself is said to be ergodic.

^c^ In a generalised PBN framework a network variable can have any value in {0,1,…,*d*−1}, where *d* > 2.

^d^ In the graph-theoretical terminology the notion of an ergodic set of states in a Markov chain corresponds to the notion of a bottom strongly connected component in a graph.

^e^ In computer science, the complexity of a function or an algorithm is expressed or characterised using the big *O* notation, namely, how the function or algorithm responds to changes in its input size.

^f^ Optimisation deals with a broad range of problems, relying on, for example, convex programming, optimal control, combinatorial optimisation or evolutionary computation paradigms; examples and additional information can be found by referring to [158-165]

^g^ A one-time gene perturbation changes the value of one or more genes without modifying the rules or probabilistic parameters of the network.

^h^ In computational complexity theory, NP-hard is a class of problems that are at least as hard as the hardest problems in NP (nondeterministic polynomial time).

## Competing interests

The authors declare that they have no competing interests.

## Authors’ contributions

PT wrote background, PBNs for the representation of biological networks, PBNs for multi-level Systems Biology, computational tools, partly of a perspective on potential applications of PBNs in clinic, and co-ordinate overall writing. AM wrote theoretical and mathematics sections on introduction, PBNs dynamic, inference and technical comparison between PBN to other probabilistic graphical models. JP wrote theoretical and mathematics sections on intervention and control of PBNs. AT wrote a part on the optimisation of PBNs. JS shared a medical perspective on the application of PBNs and wrote a perspective on potential applications of PBNs in clinic. TS supervised and integrated the overall writing together with PT and revised the manuscript. All authors read and approved the final manuscript.
